# Insecticide resistance and malaria transmission: infection rate and oocyst burden in *Culex pipiens *mosquitoes infected with *Plasmodium relictum*

**DOI:** 10.1186/1475-2875-9-379

**Published:** 2010-12-31

**Authors:** Julien Vézilier, Antoine Nicot, Sylvain Gandon, Ana Rivero

**Affiliations:** 1 Génétique et Evolution des Maladies Infectieuses (UMR CNRS 2724), Centre de Recherche IRD, 911 Avenue Agropolis, 34394 Montpellier, France; 2 Centre d'Ecologie Fonctionnelle et Evolutive (UMR CNRS 5175), 1919 Route de Mende, 34294 Montpellier, France

## Abstract

**Background:**

The control of most vectors of malaria is threatened by the spread of insecticide resistance. One factor that has been hitherto largely overlooked is the potential effects of insecticide resistance on the ability of mosquitoes to transmit malaria: are insecticide-resistant mosquitoes as good vectors of *Plasmodium *as susceptible ones? The drastic physiological changes that accompany the evolution of insecticide resistance may indeed alter the ability of vectors to transmit diseases, a possibility that, if confirmed, could have major epidemiological consequences.

**Methods:**

Using a novel experimental system consisting of the avian malaria parasite (*Plasmodium relictum*) and its natural vector (the mosquito *Culex pipiens*), two of the most common mechanisms of insecticide resistance (esterase overproduction and acetylcholinesterase modification) were investigated for their effect on mosquito infection rate and parasite burden. For this purpose two types of experiments were carried out using (i) insecticide-resistant and susceptible laboratory isogenic lines of *Cx. pipiens *and (ii) wild *Cx. pipiens *collected from a population where insecticide resistant and susceptible mosquitoes coexist in sympatry.

**Results:**

The isogenic line and wild-caught mosquito experiments were highly consistent in showing no effect of either esterase overproduction or of acetylcholinesterase modification on either the infection rate or on the oocyst burden of mosquitoes. The only determinant of these traits was blood meal size, which was similar across the different insecticide resistant categories in both experiments.

**Conclusions:**

Insecticide resistance was found to have no effect on *Plasmodium *development within the mosquito. This is the first time this question has been addressed using a natural mosquito-*Plasmodium *combination, while taking care to standardize the genetic background against which the insecticide resistance genes operate. Infection rate and oocyst burden are but two of the factors that determine the vectorial capacity of mosquitoes. Other key determinants of parasite transmission, such as mosquito longevity and behaviour, or the parasite's incubation time, need to be investigated before concluding on whether insecticide resistance influences the ability of mosquitoes to transmit malaria.

## Background

Many of the most dangerous human diseases are transmitted by mosquitoes. Insecticide use is the mainstay of mosquito control programmes [1], and insecticide resistance one of its biggest obstacles [2]. Insecticide resistance jeopardizes disease control efforts by increasing the number of mosquitoes that survive the insecticide treatment and are available to spread the parasite in the population. Although this *quantitative *effect of insecticide resistance on the mosquitoes may be mitigated by the costs associated to insecticide resistance [3-6] it has been deemed sufficiently worrying to motivate the development of resistance management strategies to prevent or retard the spread of resistance [7-9]. Little attention has however been given to the potential *qualitative *effects of insecticide resistance on the mosquitoes: are insecticide resistant mosquitoes better, equal or worse vectors of diseases than susceptible ones? Mosquitoes are not mere flying syringes, they provide a very specific physiological environment in which parasites differentiate, proliferate and migrate to the correct tissues to ensure transmission to the next host. There is increasing evidence that this environment is drastically modified when insects become resistant to insecticides [10]. For instance, recent work by McCarroll and co-workers [11,12] has shown that insecticide resistance levels in wild *Culex quinquefasciatus *mosquitoes are negatively correlated with the density of the filarial parasite *Wuchereria bancrofti*, and that parasite development is blocked at the L1 stage in laboratory mosquitoes selected for artificially high levels of insecticide resistance [11,13]). These results could extend to other mosquito-parasite combinations and have large implications for the transmission of diseases.

Malaria is one of the most lethal diseases of humans. It is caused by protozoa of the genus *Plasmodium *that parasitize mammals, birds and lizards. Mosquito control through the use of insecticides is still the most important component of malaria control programmes. Insecticide resistance, including multiple resistance to all the major classes of insecticides, has been reported in all the main mosquito vectors of malaria [14]. Mosquitoes are able to escape the lethal effects of insecticides by two non exclusive physiological strategies: by reducing the sensitivity of the neural targets of the insecticides (target site resistance) and by increasing the activity of detoxifying enzymes (metabolic resistance [2]). In some areas, the prevalence of these types of insecticide resistance is so high - upwards of 50% [15,16] - that *Plasmodium *parasites circulating in the blood of infected hosts have a high chance of being ingested by an insecticide resistant mosquito. Despite this, surprisingly little is known as to whether insecticide resistance interferes with the subsequent development of *Plasmodium *within the vector. This important gap in our knowledge is partly due to the difficulties associated with finding sympatric insecticide-resistant and susceptible mosquitoes, particularly in areas with a long and complex history of insecticide use where multiple resistance mechanisms are the norm [17] and fully susceptible individuals hard to find. This has driven researchers to work on allopatric mosquito combinations [18,19] or on mosquito-*Plasmodium *combinations not found in nature [20], both of which render the results difficult to interpret [10].

This study investigates the potential effect of insecticide resistance on *Plasmodium *infection rate and parasite burden within mosquitoes using the avian malaria parasite, *Plasmodium relictum*, and its natural mosquito vector, *Culex pipiens *[21]. Avian malaria parasites share a distant common ancestor with human malaria parasites [22] and have historically played an important role as models in the study of human malaria [23]. *Culex pipiens *is one of the main vectors of avian *Plasmodium *[21] and is widely present in the south of France, where it is seen as a nuisance to the tourist industry. Following repeated treatments of larval sites with organophosphate insecticides (initiated 40 years ago), the two main types of insecticide resistance are present in the Montpellier region: target site resistance (through the modification of the acetylcholinesterase [24]) and metabolic resistance (through the overproduction of detoxifying carboxylesterases [25]). One particularly convenient feature of this system is that mosquito control has been limited to the populations along the Mediterranean coast. It is thus possible to identify an insecticide-treated area (a 20 Km band close to the sea), a non-treated area (further north), and an intermediate area where metabolic and target site resistant mosquitoes coexist with susceptible ones [26]. In addition, through a series of back-crossings carried out at the Institut des Sciences de l'Evolution de Montpellier, the different insecticide resistance alleles found in the region have been separately introgressed into a common (insecticide-susceptible) genetic background to produce different isogenic insecticide-resistant mosquito lines [4].

Qualitatively different predictions can be made on the effect of the two different insecticide resistant mechanisms on *Plasmodium *development within the mosquito. These have been extensively reviewed elsewhere [10] and will only be briefly outlined here. First, the overproduction of large amounts of detoxifying esterases results in a substantial depletion of the energetic stores of mosquitoes [27]. Resource depletion can have two contrasting consequences for parasite development: on the one hand it may hinder mosquito immunity, whose maintenance and deployment are known to be resource dependent [28], thereby favoring parasite development. On the other hand, it may limit the development of *Plasmodium*, a parasite whose mosquito stages are known to be greedy consumers of resources [29,30]. Second, esterases have been shown to be highly expressed in mosquito tissues that are key for parasite development (such as the midgut [31]). These overproduced esterases may render these tissues toxic for parasite development through, amongst others, an excess production of reactive oxygen species - a possibility that has never been formally explored but for which there is indirect evidence [10,11]. Finally, acetylcholinesterase modification has been shown to increase the feeding rate of *Cx. pipiens *larvae [32], most likely as a consequence of an hyperactive nervous system resulting from an excess of acetylcholinesterase in the synapses [3]. Hyperactivity may also have an impact on the blood feeding efficiency of the adults and thus on the number of parasites ingested, though not necessarily on their subsequent development [10].

This paper aims to reply to the following three questions: 1) Do insecticide resistant mosquitoes have a different probability of infection than susceptible ones? 2) Do infected insecticide resistant mosquitoes attain higher or lower parasite burdens than susceptible ones? and 3) Are these differences dependent on the underlying insecticide resistance mechanism (metabolic vs target site)? For this purpose, experimental *P. relictum *infections were carried out using (i) insecticide-resistant and susceptible laboratory isogenic lines of *Cx. pipiens *and (ii) wild *Cx. pipiens *collected from a population where insecticide resistant and susceptible mosquitoes coexist in sympatry. The isogenic lines allowed us to test the effect of the insecticide resistance genes in a uniform genetic background. This may increase the chances of detecting an eventual pleiotropic effect of the insecticide resistance genes, but the results may not be necessarily applicable to other genetic backgrounds, particularly if there are epistatic interactions between the insecticide resistant genes and other genes in the genome (although modifier genes have not yet been described in this species [33]). The field-collected mosquitoes, on the other hand, allowed us to test the effects under the more realistic conditions of a heterogeneous genetic background [34]. Combined, these two approaches provide a powerful test of the role of insecticide resistance on parasite development within the mosquito.

## Methods

### Avian malaria parasite

*Plasmodium relictum *(lineage SGS1) is the aetiological agent of the most prevalent form of avian malaria in Europe [21]. This generalist *Plasmodium *parasite lineage was originally isolated by G. Sorci (CNRS, Dijon) from wild sparrows caught in the region of Dijon (France) in 2008 (wild mosquito experiments) and 2009 (isogenic line experiments) and subsequently passaged to naïve canaries (*Serinus canaria*) by intra peritoneal injection. The strain was maintained in an animal house by carrying out regular passages between stock canaries every ca. 3 weeks. At the time of the experiments, the parasite had undergone between nine (wild mosquito experiments) and fourteen (isogenic line experiments) passages since their transfer from the sparrows. For the purpose of the experiments, the experimental canaries were infected by intra-peritoneal injection of ca. 50-100 μL of blood from the infected canary stock. Their parasitaemia was regularly monitored from the fifth day of infection onwards using thin blood smears as described in [21]. Mosquito feeding (see below) took place 10 days after the onset of the infection, to coincide with the acute phase of the parasitaemia (Vézilier, unpublished results).

### Isogenic strain experiments

#### Mosquito rearing

Experiments were carried out using one insecticide susceptible strain (SLAB), two insecticide resistant strains through the overproduction of detoxifying esterases (SA2B2, SA4B4) and one strain with an insensitive acetylcholinesterase but no overproduced esterases (SR). Details of these strains are given in Table 1. Eggs of each of the different mosquito strains were obtained from the Institut des Sciences de l'Evolution de Montpellier and set up to hatch under standard insectary conditions (25 ± 1°C, 70 ± 5% RH and 12L: 12D photoperiod). On the hatching day, larvae were haphazardly seeded into plastic trays (4 trays per genotype, dimensions: 25 cm × 35 cm × 7 cm) containing one litre of mineral water (Eau de Source Carrefour, France) at a constant density of 300 individuals per tray. Larvae were provided with a half-tablet of concentrated yeast on the day of the hatching, 200 mg of TetraMin^® ^fish flakes the following day, and from then on 400 mg TetraMin every two days until pupation. Tray water was changed on feeding days to avoid bacterial growth on the water surface. On pupation, trays were placed inside an emergence cage (27 × 40 × 35 cm) and provided with an *ad libitum *source of 10% sugar solution for the emerged adults.

**Table 1:** Insecticide resistant and susceptible strains used in the isogenic strain experiment.

Strain	IR mechanism	Alleles	Genetic background
**SLAB**	None	*Ester*^0^, *ace-1*^S^	SLAB
**SA2B2**	Overproduction of esterases A2 and B2	*Ester*^2^, *ace-1*^S^	SLAB
**SA4B4**	Overproduction of esterases A4 and B4	*Ester*^4^, *ace-1*^S^	SLAB
**SR**	Insensitive acetylcholinesterase	*Ester*^0^, *ace-1*^R^	SLAB

The overproduction of esterases is controlled by a superlocus consisting of two loci (*esterase A *and *esterase B*) in complete linkage disequilibrium. Alleles for this locus are the wild type susceptible *Ester*^0^, or the insecticide resistant *Ester^2 ^*(overproduces the esterase A2 and B2 isozymes) and *Ester^4 ^*(overproduces the esterase A4 and B4 isozymes). The modification of the acetylcholinesterase is controlled by the locus *ace-*1. Alleles for this locus are the wild type susceptible *ace-1^S ^*and the insecticide resistant *ace-1^R ^*(which contains a single GGC→AGC point mutation that renders the acetylcholinesterase insensitive to the insecticide). For more details on those strains, see [4].

#### Mosquito experimental infections and dissections

One day before the feeding, 50 female mosquitoes from each of the four strains (SLAB, SA4B4, SA2B2, SR) were haphazardly chosen from the different emergence cages and placed inside an experimental cage. Three such experimental cages were obtained in this way. The mosquitoes in these experimental cages were deprived of glucose for 24 h to increase hunger levels and thus favour blood feeding. On the morning of the blood feeding day, bird parasitaemia was estimated by counting the proportion of infected red blood cells using Giemsa-stained blood smears. Blood feeding was carried out overnight by placing a different experimental *Plasmodium*-infected canary inside an upturned aerated containment box (diameter 14 cm, height 12 cm) on top of each cage. The canaries stood on top of the cage allowing the mosquitoes to feed on the bird's feet through the screened wall, while the rest of the body was protected from the bites. Engorged females were taken out from the cages, briefly anesthetized with CO_2 _and placed individually into numbered dry 30 ml drosophila plastic tubes covered with a mesh. Food was provided in the form of a cotton pad soaked in a 10% glucose solution placed on top of each tube. This cotton pad was replaced daily throughout the remainder of the experiment.

Due to the high number of replications and the time required to dissect out and count oocysts in the mosquito gut, mosquito dissections were spread out over four consecutive days: days 5-8 post blood meal (pbm). Each dissection day, one fourth of the blood-fed mosquitoes that had fed on each of the canaries were haphazardly chosen, taken out from their tubes and dissected under a binocular microscope in 100 μl of 0.01 M phosphate-buffered saline (PBS). The tubes were kept at 4°C for haematin analysis (see below). The dissected midguts were transferred with a pin to a slide containing a drop of PBS with 5% mercurochrome. The slide was observed under a phase contrast microscope equipped with a 40x oil immersion objective to assess infection rate (oocysts present/absent) and oocyst burden (number of oocysts in infected guts). Dissected bodies were kept on ice immediately after dissection and subsequently frozen at -80°C for genotype identification. Mosquito genotype (SLAB, SA2B2, SA4B4 or SR) was determined using RFLP analysis as described in [35,36]. Quantification of the haematin (a by-product of the decomposition of haemoglobin) excreted at the bottom of the tubes was carried out as described in previous papers [37]. The purpose of haematin quantification is to correct for potential differences in the amount of blood ingested by females of different genotypes. Solutions with an absorbance ≤ 0.01 were classified as being from mosquitoes that had not blood fed, as this absorbance was indistinguishable from the LiCO_3 _control.

### Wild mosquito experiments

#### Mosquito collections and rearing

Wild *Cx. pipiens *mosquito larvae were collected in August (Block 1), September (Block 2) and October (Block 3) 2008 from a sympatric population of insecticide resistant and susceptible mosquitoes. This population was found 20 km north of Montpellier, in a sewage treatment lagoon (near the village of Triadou, France) located at the boundary between an insecticide-treated and non-treated zone [33]. A previous sampling of the larvae in this basin carried out in June of the same year had given a balanced proportion of each of the different insecticide resistant genotypes (data not shown). The larvae collected were brought to the insectary, sorted by developmental stage, and seeded haphazardly in five plastic trays at a constant density of 300 individuals per tray. The rearing conditions were identical to those used for the isogenic strains (see above).

Two additional trays were seeded with 300 larvae of the SLAB laboratory strain of *Cx. pipiens *(see above). The SLAB mosquitoes were reared in parallel and in identical (density, food, insectary) conditions to the wild collected mosquitoes. To discriminate SLAB from wild mosquitoes, four days before the experiment, SLAB females were marked with a RadGlo^® ^JST fluorescent pigment applied using a dust storm technique as described in [38]. The amount of dust applied was 50 μg for 50 individuals, which in preliminary trials was found to have no effect on mosquito survival or oocyst count (Flore Zélé and Julien Vézilier, unpublished data), and was only detectable using a binocular microscope.

#### Mosquito experimental infection and dissections

Experimental infections were carried out in identical way to the isogenic strain experiments (see above). Each cage (3 cages per block) contained 150 wild female mosquitoes and 50 marked SLAB mosquitoes, haphazardly chosen from the emergence cages. A different infected canary was placed overnight on top of each of the cages for the blood feeding to take place. The presence of SLAB mosquitoes in the cages served a double purpose: on the one hand, to obtain a standard measure of parasite infectivity to mosquitoes in each of the cages. On the other hand it allowed us to compare the infectivity of *P. relictum *in wild-caught vs. lab-reared isogenic lines.

As above, mosquito dissections were spread out over 4 consecutive days: days 5-8 post blood meal (pbm). Each day, one fourth of SLAB and wild mosquitoes (n = 12 SLAB and n = 25 wild mosquitoes) from each cage were haphazardly chosen, taken out from their tubes and the measurements of infection rate, oocyst burden, and haematin quantification obtained as for the isogenic strain mosquitoes. Wild mosquitoes were typed to determine their insecticide status in the following ways. Insecticide resistance through modification of the acetylcholinesterase was established by analysing homogenates of the heads of individual mosquitoes using the micro-plate TPP test [39]. Insecticide resistance through the overproduction of carboxylesterases was analysed on single mosquito thorax homogenates by starch-gel electrophoresis in TME 7.4 buffer systems [40]. This technique allows us to distinguish between the three main different insecticide resistant carboxylesterase allozymes present in the study area: A1 (allele *Ester^1^*), A4-B4 (allele *Ester^4^*) and A2-B2 (allele *Ester^2^*)[25]. Because of haemolymph loss (and subsequent active esterase loss) during the dissection process, some thorax homogenates didn't allow us to establish the carboxylesterase resistance status through starch-gel electrophoresis. In these cases (n = 176), the abdomen homogenates were analysed using RFLP analysis (as above). Once typed, field collected mosquitoes were allocated to one of 4 insecticide resistance status: S (fully susceptible), E (resistant through esterase overproduction), A (resistant through acetylcholinesterase modification) or AE (resistant through both acetylcholinesterase modification and esterase overproduction, see Table 2 for details).

**Table 2:** Number of mosquitoes from the different insecticide resistant categories used in the wild mosquito experiments.

IR status	Alleles	Block 1	Block 2	Block 3
S	*Ester*^0^, *ace-1*^S^	83 (30.07%)	88 (27.16%)	90 (27.78%)
				
E	*Ester*^1^, *ace-1*^S^	2 (0.72%)	7 (2.16%)	4 (1.23%)
	*Ester*^2^, *ace-1*^S^	7 (2.54%)	3 (0.93%)	2 (0.62%)
	*Ester*^4^, *ace-1*^S^	77 (27.9%)	98 (30.25%)	86 (26.55%)
				
A	*Ester*^0^, *ace-1*^R^	42 (15.22%)	54 (16.67%)	62 (19.13%)
				
AE	*Ester*^1^, *ace-1*^R^	2 (0.72%)	3 (0.92%)	6 (1.85%)
	*Ester*^2^, *ace-1*^R^	4 (1.45%)	2 (0.62%)	3 (0.93%)
	*Ester*^4^, *ace-1*^R^	59 (21.38%)	69 (21.29%)	71 (21.91%)
				
**Total**		**276 (100%)**	**324 (100%)**	**324 (100%)**

The corresponding proportions are given in brackets. The different insecticide resistance status are S: fully susceptible, E: resistance through esterase overproduction, A: resistant through acetylcholinesterase modification and AE: resistant through both esterase overproduction and AChE modification.

All experiments described above were conducted in accordance with the French Government regulations for animal experimentation. Canaries were housed in a licensed animal house (Direction Départementale des Services Vétérinaires de l'Hérault - DDSV, licence number: E 34-172-21). A. Rivero is authorized under French law to experiment on canaries (DDSV authorization number: 34.363).

### Statistical analyses

The analyses were carried out using the R statistical package (v.2.10.1). The different statistical models applied to the data are described in Additional File 1. The analysis of infection rate (oocyst presence/absence) was carried out using the *lmer *mixed model procedure with binomial errors (lme4 package). For the analysis of infection intensity (number of oocysts), only individuals that developed ≥ 1 oocyst were included. As has been found in other systems [41,42] oocyst count data were greatly overdispersed. One way of handling this overdispersion is by using negative binomial pseudo distributions [42]. However, to date, it is not currently possible to account for negative binomial distributions within a mixed model *lmer *procedure. For this reason, data were Box-Cox transformed and analysed using the *lme *mixed model procedure (nlme package). For the graphical analysis of the relationship between oocyst burden and haematin (individuals that developed ≥ 1 oocyst), the *gam *procedure was used (mgcv package).

The insecticide resistance status of the insects (SLAB, SA4B4, SA2B2, SR in the isogenic strain experiment and S, E, A, AE in the wild mosquito experiments), and the amount of haematin (the standard proxy for blood meal size [43]) were fitted as fixed explanatory variables. Maximal models were simplified by sequentially eliminating non-significant terms and interactions to obtain a minimal model [44]. The significance of the explanatory variables was established using a likelihood ratio test (LRT) which is approximately distributed as a χ^2 ^distribution [45]. The significant χ^2 ^values given in the text are for the minimal model, while non-significant values correspond to those obtained before deletion of the variable from the model. When appropriate, such as in the analysis of the significant effect of insecticide resistance status in block 3 of the wild mosquito experiments, *a posteriori *contrasts were carried out by aggregating factor levels that did not significantly differ from each other and by testing the fit of the simplified model using an LRT [44]. As oocyst burden could be expected to be a non-linear function of haematin quantity, the quadratic term haematin^2 ^was added to the minimal model to assess if it significantly improved the model fit. Differences between birds and dissection days were often significant but as they are of no interest in their own right, they were controlled for by leaving these terms as random factors in the model (see Additional File 1). The one exception was in the comparison of SLAB and wild-caught mosquitoes (models 19 and 20) where dissection day had to be fitted as a fixed factor rather than a random factor, as the latter did not allow for model convergence (dissection day was however not retained in the minimal model). The three blocks of the wild mosquito experiments, which were obtained at three different monthly intervals, were first analysed separately (one analysis per block). A second, more stringent, test was subsequently carried out by analyzing all blocks together and adding block as an additional random factor to the model.

## Results

### Isogenic strain experiments

After overnight exposure with the infected canaries, 85% of the SLAB, 88% of the SA4B4, 94% of the SA2B2 and 85% of the SR mosquitoes had taken a blood meal and were subsequently dissected for oocyst detection. The rest either died before feeding or did not blood feed.

**Figure 1:**
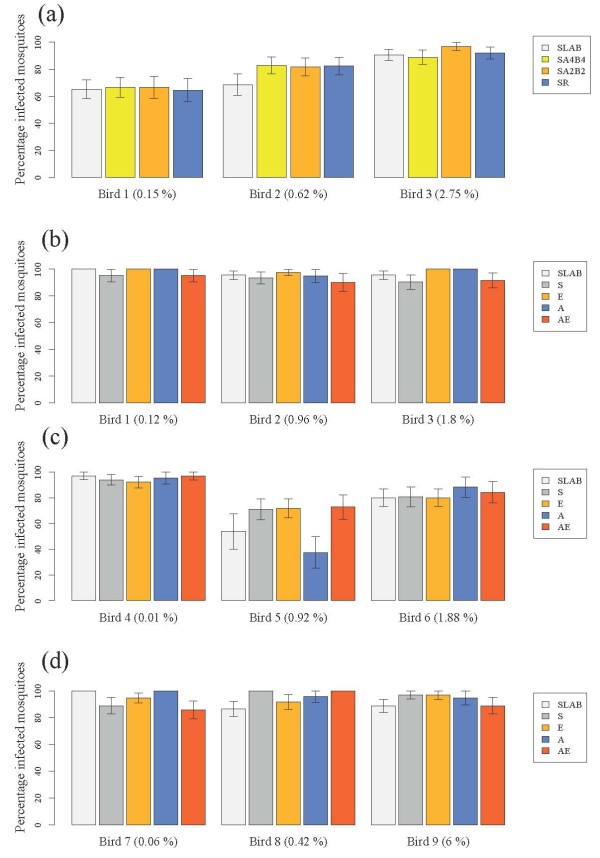
**Infection rate of insecticide-resistant and susceptible mosquitoes in (a) the isogenic strain experiment and (b) block 1, (c) block 2 and (d) block 3 of the wild mosquito experiments**. Three different experimentally infected birds were used in each of the four experiments (bird parasitaemia at the day of the feed is indicated in brackets). The figure shows the mean (± se) proportion of mosquitoes with at least one oocyst in. See Tables 1 and 2 for details of mosquitoes used in each experiment.

**Figure 2:**
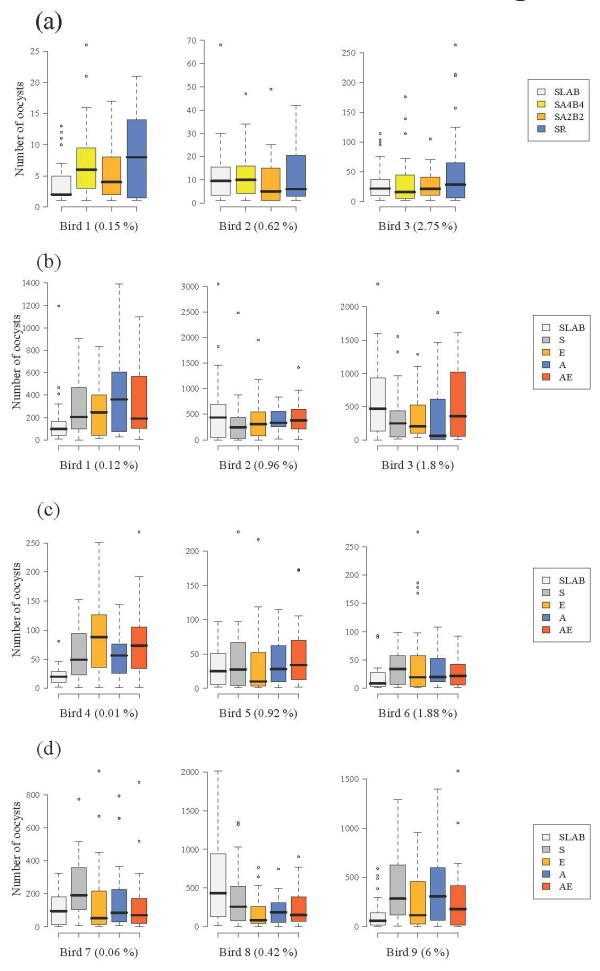
**Oocyst burden of insecticide resistant and susceptible mosquitoes in (a) the isogenic strain experiment and (b) block 1, (c) block 2 and (d) block 3 of the wild mosquito experiments**. Three different experimentally infected birds were used in each of the four experiments (bird parasitaemia at the day of the feed is indicated in brackets). The figure shows the median number of oocysts (horizontal black bars). The coloured boxes below and above the median indicate the first and third quartiles respectively. Dashed lines delimit 1.5 times the inter-quartile range on both side of the box, above which individual counts are considered outliers and marked as dots. See Tables 1 and 2 for details of mosquitoes used in each experiment.

**Figure 3:**
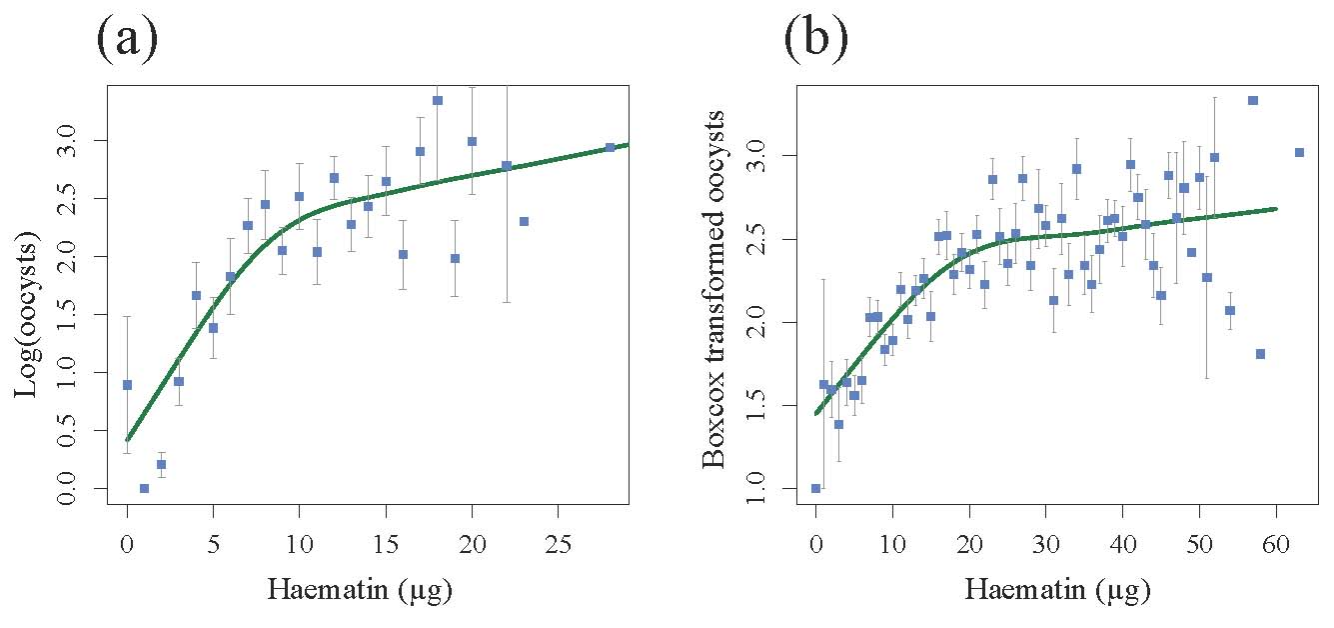
**Relationship between number of oocysts and blood meal size in (a) the isogenic strain experiment and (b) cumulative dataset for the three wild mosquito experiments**. General additive models were fitted to each dataset using all mosquito replicates independently of their insecticide resistant status. Squares represent the mean number of oocysts for each haematin value (haematin values were rounded up to the nearest integer). Bars above and below the means represent the standard errors of the mean. The fitted curve corresponds to the predicted values arising from the statistical models.

There were no significant differences between the insecticide resistant and susceptible strains in either blood meal size (model 1, χ^2^_3_= 1.56, p = 0.66), probability of infection (model 2, χ^2^_3 _= 1.70, p = 0.63, Figure 1a) or oocyst burden (model 3, χ^2^_3 _= 6.90, p = 0.08, Figure 2a). The probability of becoming infected with *P. relictum *and the amount of oocysts that successfully developed in the mosquito increased drastically with the amount of blood consumed (model 2, χ^2^_1 _= 97.5, p < 0.0001 and model 3, χ^2^_1 _= 112.55, p < 0.0001, respectively), irrespective of the strain. Fitting the quadratic term (haematin^2^) highly improved the model fit (model 3, χ^2^_1 _= 24.95, p < 0.0001), suggesting that oocyst burden was a decelerating polynomial function of blood meal size (Figure 3a).

### Wild mosquito experiments

The number of field-collected mosquitoes from each allelic variant that took a blood meal and were subsequently dissected for oocyst detection is given in Table 2. The oocystaemias observed in the wild mosquito experiment were, on average, significantly larger than those found in the isogenic strain experiment (mean ± s.e., 208 ± 10 and 16 ± 1 respectively, all strains combined, χ^2^_1 _= 5.46, p = 0.02). This turned out to be an experiment effect, rather than a laboratory vs field mosquito effect, as the comparison of SLAB vs field-caught mosquitoes within the wild mosquito experiment showed no significant differences in either infection probability (model 19: χ^2^_1 _= 0.0084, p = 0.93, Figure 1b-d), or oocyst burden (model 20: χ^2^_1 _= 1.93, p = 0.16, Figure 2b-d).

The effect of insecticide resistance status on blood meal size, infection probability and oocyst burden was first analysed separately for each block. For the first two blocks, blood meal size was independent of the insecticide resistant status of the mosquitoes (Block 1, model 4: χ^2^_3 _= 4.10, p = 0.25; Block 2, model 8: χ^2^_3 _= 4.31, p = 0.23). In block 3, however, S females ingested slightly more blood than the other insecticide resistant categories (model 12, χ^2^_3 _= 9.54, p = 0.023). In all three blocks the infection rate was strongly dependent on the amount of haematin ingested (Block 1, model 5: χ^2^_1 _= 13.14, p = 0.0003; Block 2, model 9: χ^2^_1 _= 33.38, p < 0.0001; Block 3, model 13: χ^2^_1 _= 8.13, p = 0.0043) but independent of the insecticide resistance status of the mosquitoes (Block 1, model 5: χ^2^_3 _= 4.11, p = 0.25; Block 2, model 9: χ^2^_3 _= 1.73, p = 0.63; Block 3, model 13: χ^2^_3 _= 1.44, p = 0.69, Figure 1b-d). Oocyst burden was also strongly positively correlated to the amount of haematin ingested (Block 1, model 6: χ^2^_1 _= 74.57, p < 0.0001; Block 2, model 10: χ^2^_1 _= 27.89, p < 0.0001; Block 3, model 14: χ^2^_1 _= 49.00, p < 0.0001). Adding the quadratic term (haematin^2^) significantly improved the model fit in two blocks out of three (Block 1, model 6: χ^2^_1 _= 6.30, p = 0.01; Block 2, model 10: χ^2^_1 _= 8.79, p = 0.003, Block 3, model 14: χ^2^_1 _= 2.58, p = 0.11). The insecticide resistance status of mosquitoes was not a significant explanatory factor of oocyst burden in blocks 1 (model 6, χ^2^_3 _= 3.85, p = 0.28, Figure 2b) and 2 (model 10, χ^2^_3 _= 1.01, p = 0.80, Figure 2c), but became significant in block 3 (model 14, χ^2^_3 _= 10.50, p = 0.01, Figure 2d). In this block, insecticide resistant mosquitoes had significantly fewer oocysts than the susceptible ones (Block 3, model 14: χ^2^_1 _= 7.49, p = 0.006), independently of the insecticide resistance mechanism involved (Block 3, model 14: χ^2^_2 _= 3.02, p = 0.22).

Analysing the three blocks together did not alter the results for infection rate, which was still independent of insecticide resistance status (model 16: χ^2^_3 _= 0.79, p = 0.85). The significant effect of insecticide resistance on oocyst burden, however, disappeared (model 17: χ^2^_3 _= 2.76, p = 0.43), leaving only haematin and haematin^2 ^as explanatory variables in the model (Figure 3b). Using the mean number of oocysts in SLAB mosquitoes as a standard measure of parasite infectivity did not alter any of the above results (models 7, 11, 15 and 18).

## Discussion

An extensive investigation into the effect of two main mechanisms of insecticide resistance on the infection rate and parasite burden of *P. relictum *infections was carried out by means of a series of experiments using both isogenic mosquito lines and field-collected mosquitoes. Infection rate determines the proportion of infected mosquitoes in a population and is a key parameter in models of malaria transmission [46]. Oocyst burden is directly related to the number of transmissible (sporozoites) stages that subsequently develop in the mosquito salivary glands [41] and has been recently shown to be strongly correlated with mosquito longevity, a key component of the mosquito's vectorial capacity [47].

It has been suggested that different mechanisms of insecticide resistance could alter both of these parameters [10], and similar insecticide resistant mechanisms have been shown to alter the development of other parasites [11,12]. However, aside from a marginal, albeit statistically significant, reduction in oocyst burden in insecticide resistant insects within the third block of the wild mosquito experiments, no clear effect of either esterase overproduction or of acetylcholinesterase modification on either infection rate (proportion of mosquitoes containing at least one oocyst) or oocyst burden was found. The isogenic line and wild-caught mosquito experiments were consistent in showing that the only determinant of infection rate and oocyst burden was blood meal size, which was similar across the different insecticide resistant categories in both experiments. The number of oocysts in the midgut of infected mosquitoes was found to be a saturating function of blood meal size: as the amount of blood ingested increases, the number of ookinetes that successfully encyst in the mosquito midgut reaches a limit. Provided that blood meal size was directly proportional to the amount of parasites ingested, these results seem to be in accordance with a recent study showing the existence of a saturating gametocyte-ookinete and/or ookinete - oocyst transition associated to high parasite densities [41].

There are two potential explanations for the lack of insecticide resistance effects on infection rate and oocyst burden. The first explanation is that, contrary to predictions, the physiological alterations associated to insecticide resistance are not sufficiently important to alter the development of *P. relictum *within the mosquito. Vontas *et al *[20] also failed to show differences in parasite burden when comparing a pyrethroid-resistant and a susceptible strain of *Anopheles stephensi *infected by *Plasmodium yoelii*. Their results were however difficult to interpret for two reasons. Firstly, because the insecticide resistant and susceptible strains had different geographic origins (the resistant DUB-R strain originated from Dubai in 1986, while the susceptible BEECH strain originated from India in the 1940s). The genetic background differences that inevitably arise during such a divergent evolutionary history could have potentially blurred the pleiotropic effects of insecticide resistant genes on *Plasmodium *development. Secondly, because *An. stephensi *is not a natural vector for *P. yoelii*. Lessons learned from immune [48,49] and longevity [50] studies, have taught us that natural and unnatural mosquito-*Plasmodium *combinations can render substantially different results (see also [34,51,52]). The present study overcomes this two potential pitfalls by using a natural vector-mosquito combination [21] and by carrying the experiments using both isogenic mosquito strains and sympatric wild-caught mosquitoes.

The second potential explanation is that the high oocystaemias obtained in the experiments may have swamped any eventual physiological differences existent between insecticide resistant and susceptible mosquitoes. Oocystaemias within the range of those found in this study are a common outcome when human malaria vectors (*Anopheles sp*) are experimentally infected with rodent malaria parasites [41,47,53] and contrast sharply with the low oocystaemias found in *Anopheles *mosquitoes caught in endemic human malaria areas [51]. Part of the reason may lie in the novel nature of such experimental host-parasite associations, which can result in increased parasite virulence [34,51]. To date, no studies have investigated *P. relictum *oocystaemias in wild-caught mosquitoes, but it is safe to assume that, despite being a natural mosquito-parasite combination, the oocystaemias obtained in this study were unnaturally high. One likely explanation for these high oocystaemias is the high bird parasitaemia at the time of the blood feed [54]. To maximize infection success, most experimental mosquito infections, and the ones here were no exception, are carried out by feeding mosquitoes on hosts at the peak of their parasitaemia, a situation unlikely to be encountered by most blood feeding mosquitoes in the field. The large majority of birds in the field are survivors of past acute infections and have very low chronic parasitaemia [21,55-57]. Could the power to detect differences between insecticide resistant and susceptible mosquitoes have been increased had the mosquitoes had lower oocystaemias? This question could be resolved by feeding mosquitoes on chronically infected birds, which recent pilot studies have shown to render significantly lower infection rates and oocyst burdens (S. Cornet pers. com).

A marginal, albeit statistically significant, reduction of oocyst numbers in insecticide resistant mosquitoes (irrespective of the mechanism) was found in the third block of the wild-caught mosquito experiment. This result is intriguing as it goes in the expected direction of a reduction in parasite numbers associated with insecticide resistance [10-12]. The signal is however weak; on closer look, the effect only appears in two out of the three birds, and in only one of the blocks. Further experiments using chronic infections are needed before it can be determined whether this result was due to a statistical type I error (in other words, a false positive) or whether there is a real biological phenomenon underlying it.

## Conclusions

Esterase and acetylcholinesterase-based insecticide resistance did not have a clear effect on the infection rate or oocyst burden in *Cx pipiens *mosquitoes, at least under the specific experimental conditions detailed above. Other key determinants of disease transmission, such as mosquito longevity and behaviour, or the parasite's incubation time within the mosquito, need to be investigated before concluding on the effects of these two mechanisms of insecticide resistance on the ability of these mosquitoes to transmit malaria [10]. The *Cx. pipiens - P. relictum *system provides a good opportunity for investigating this question, not least because it is the only currently available non-human experimental model that uses a natural mosquito-*Plasmodium *combination and allows us to standardize the genetic background against which the insecticide resistant genes operate. The congruency of the results obtained between the isogenic strain and wild-caught mosquito experiments further suggests that, at least for the variables measured, the laboratory strains are not too far removed from field-caught mosquitoes, an additional advantage for experimental purposes.

The ultimate question is, however, whether insecticide resistance affects human malaria transmission, as this can have important public health consequences. The long and complex history of insecticide use in most endemic malarial areas greatly complicates the task of finding fully susceptible *Anopheles *individuals and therefore of making meaningful sympatric comparisons. The best current alternative is the establishment of isogenic lines of mosquitoes, though this option is lengthy to implement and not devoid of potential pitfalls [10,13]. Before these experimental challenges are overcome, research on animal malaria models remains a good alternative to understand the effect of insecticide resistance on the ability of mosquitoes to transmit malaria.

## Competing interests

The authors declare that they have no competing interests.

## Authors' contributions

JV, AR and SG conceived and designed the experiments. JV, AR and AN performed the experiments. AN and JV carried out mosquitoes genotyping. JV, AR and SG wrote the paper. All authors read and approved the manuscript.

## Supplementary Material

Additional file 1 **Description of statistical models used to analyse the influence of insecticide resistance on *Cx. pipiens *infection**. Click here for file
