# Supplementary material for: Insecticide resistance and malaria transmission: infection rate and oocyst burden in Culex pipiens mosquitoes infected with Plasmodium relictum
Source: Malar J. 2010 Dec 31;9:379. doi: 10.1186/1475-2875-9-379 (PMC3313086; doi:10.1186/1475-2875-9-379)
Supplement: Additional file 1 — Description of statistical models used to analyse the influence of insecticide resistance on Cx. pipiens infection. [file 1475-2875-9-379-S1.doc]

**Additional File 1: Description of statistical models used to analyse the influence of insecticide resistance on *Cx. pipiens* infection.** The response variable was not transformed unless otherwise stated (superscript indicates λ in boxcox transformation). N gives the number of mosquitoes included in each analysis. "Maximal model" gives the complete set of explanatory variables (and their interactions) included in the model. "Minimal model" gives the model containing only the significant variables and their interactions. Round brackets indicate variables fitted as random factors (nested variables are indicated with a backslash). Square brackets indicate the error structure used (n: normal errors, b: binomial errors). dd: dissection day, ir: insecticide resistance status (SLAB, SA4B4, SA2B2, SR in the isogenic strain experiment; S, E, A, AE in the wild mosquito experiment), hm: haematin excreted (blood meal size), mo: mosquito origin (SLAB vs wild caught mosquitoes).

| **Variable of interest** | **Resp.**  **variable** | **Model**  **Nb.** | **N** | **Maximal model** | **Minimal model** | **R subroutine**  **[err struct.]** |
| --- | --- | --- | --- | --- | --- | --- |
| ***Isogenic strain mosquitoes*** | |  |  |  |  |  |
| Blood meal size | hm | 1 | 449 | ir + (bird) | 1 + (bird) | lme [n] |
| Prob. infection | inf | 2 | 449 | ir* hm + (bird/dd) | hm + (bird/dd) | lmer [b] |
| Oocyst burden | log (oob) | 3 | 355 | ir* hm + hm^2^ (bird/dd) | hm + hm^2^ + (bird/dd) | lme [n] |
| ***Wild-caught mosquitoes*** | |  |  |  |  |  |
| *Block 1* |  |  |  |  |  |  |
| Blood meal size | hm | 4 | 276 | ir + (bird) | 1 + (bird) | lme [n] |
| Prob. infection | inf | 5 | 276 | ir* hm + (bird/dd) | hm + (bird) | lmer [b] |
| Oocyst burden | oob^0.27^ | 6 | 263 | ir* hm + hm^2^ (bird/dd) | hm + hm^2^ + (bird) | lme [n] |
| Normalised Oocyst burden | (oob/SLAB)^0.27^ | 7 | 263 | ir* hm + hm^2^ + (bird/dd) | hm + hm^2^ + (bird) | lme [n] |
| *Block 2* |  |  |  |  |  |  |
| Blood meal size | hm | 8 | 324 | ir + (bird) | 1 + (bird) | lme [n] |
| Prob. infection | inf | 9 | 324 | ir* hm + (bird/dd) | hm + (bird) | lmer [b] |
| Oocyst burden | oob^0.26^ | 10 | 264 | ir* hm + hm^2^ + (bird/dd) | hm + hm^2^ + (bird) | lme [n] |
| Normalised Oocyst burden | (oob/SLAB)^0.26^ | 11 | 264 | ir* hm + hm^2^ + (bird/dd) | hm + hm^2^ + (bird) | lme [n] |
| *Block 3* |  |  |  |  |  |  |
| Blood meal size | hm | 12 | 324 | ir + (bird) | ir + (bird) | lme [n] |
| Prob. infection | inf | 13 | 324 | ir* hm + (bird/dd) | hm + (bird) | lmer [b] |
| Oocyst burden | oob^0.23^ | 14 | 306 | ir* hm + hm^2^ + (bird/dd) | ir + hm + (bird) | lme [n] |
| Normalised Oocyst burden | (oob/SLAB)^0.18^ | 15 | 306 | ir* hm + hm^2^ + (bird/dd) | ir + hm + (bird) | lme [n] |
| *All blocks* |  |  |  |  |  |  |
| Prob.infection | inf | 16 | 924 | ir* hm + (block/bird/dd) | hm + (block/bird) | lmer [b] |
| Oocyst burden | oob^0.18^ | 17 | 833 | ir* hm + hm^2^ + (block/bird/dd) | hm + hm^2^ + (block/bird) | lme [n] |
| Normalised Oocyst burden | (oob/SLAB)^0.20^ | 18 | 833 | ir* hm + hm^2^ + (block/bird/dd) | hm + hm^2^ + (block/bird/dd) | lme [n] |
| *SLAB vs Wild-caught* |  |  |  |  |  |  |
| Prob. Infection | inf | 19 | 1271 | mo* hm + ***dd + (block/bird) | hm + (block/bird) | lmer [b] |
| Oocyst burden | oob^0.14^ | 20 | 1151 | mo* hm +dd + (block/bird) | hm + (block/bird) | lme [n] |
